# Adenosine relaxation in isolated rat aortic rings and possible roles of smooth muscle K_v_ channels, K_ATP_ channels and A_2a_ receptors

**DOI:** 10.1186/s40360-016-0067-8

**Published:** 2016-05-23

**Authors:** Aryadi Arsyad, Geoffrey P. Dobson

**Affiliations:** Physiology Department, Medical Faculty, Hasanuddin University, Jl. Perintis Kemerdekaan, Km. 10, Tamalanrea, Makassar, 90213 Indonesia; Heart, Trauma and Sepsis Research Laboratory, Australian Institute of Tropical Health and Medicine, College of Medicine and Dentistry, James Cook University, 1 James Cook Drive, Queensland, 4811 Australia

**Keywords:** Rat aorta, Adenosine, Vasodilation, Endothelium, Nitric oxide, Vascular tone

## Abstract

**Background:**

An area of ongoing controversy is the role adenosine to regulate vascular tone in conduit vessels that regulate compliance, and the role of nitric oxide (NO), potassium channels and receptor subtypes involved. The aim of our study was to investigate adenosine relaxation in rat thoracic aortic rings, and the effect of inhibitors of NO, prostanoids, K_v_, K_ATP_ channels, and A_2a_ and A_2b_ receptors.

**Methods:**

Aortic rings were freshly harvested from adult male Sprague Dawley rats and equilibrated in an organ bath containing oxygenated, modified Krebs-Henseleit solution, 11 mM glucose, pH 7.4, 37 °C. Isolated rings were pre-contracted sub-maximally with 0.3 μM norepinephrine (NE), and the effect of increasing concentrations of adenosine (1 to 1000 μM) were examined. The drugs L-NAME, indomethacin, 4-aminopyridine (4-AP), glibenclamide, 5-hydroxydecanoate, ouabain, 8-(3-chlorostyryl) caffeine and PSB-0788 were examined in intact and denuded rings. Rings were tested for viability after each experiment.

**Results:**

Adenosine induced a dose-dependent, triphasic relaxation response, and the mechanical removal of the endothelium significantly deceased adenosine relaxation above 10 μM. Interestingly, endothelial removal significantly decreased the responsiveness (defined as % relaxation per μM adenosine) by two-thirds between 10 and 100 μM, but not in the lower (1–10 μM) or higher (>100 μM) ranges. In intact rings, L-NAME significantly reduced relaxation, but not indomethacin. Antagonists of voltage-dependent K_v_ (4-AP), sarcolemma K_ATP_ (glibenclamide) and mitochondrial K_ATP_ channels (5-HD) led to significant reductions in relaxation in both intact and denuded rings, with ouabain having little or no effect. Adenosine-induced relaxation appeared to involve the A_2a_ receptor, but not the A_2b_ subtype.

**Conclusions:**

It was concluded that adenosine relaxation in NE-precontracted rat aortic rings was triphasic and endothelium-dependent above 10 μM, and relaxation involved endothelial nitric oxide (not prostanoids) and a complex interplay between smooth muscle A_2a_ subtype and voltage-dependent K_v_, SarcK_ATP_ and MitoK_ATP_ channels. The possible in vivo significance of the regulation of arterial compliance to left ventricular function coupling is discussed.

## Background

Adenosine is a ubiquitous endogenous mediator that is activated in response to cellular ischemic/hypoxic/shear stress [1–5]. Adenosine exerts it cellular effects by binding to four major subtypes of the G-protein-coupled receptors; A_1_, A_2a_, A_2b_, and A_3_ which activate intracellular survival kinase pathways in a cell- and tissue-specific manner [2, 3, 5, 6]. Through receptor-modulation and downstream signaling pathways adenosine alters coronary and peripheral vascular tone, cardiac function, brain and central nervous system signalling, sleep, the state of natural hibernation, ischemic preconditioning, post-conditioning, inflammation, coagulation, angiogenesis and cell proliferation and remodelling [4–7].

An area of ongoing controversy is the role adenosine to regulate vascular tone in the arterial tree, and the receptor subtypes involved. The subtype A_2a_ appears to be the predominate receptor in arterial vasodilation in mouse, rat, guinea pig, pigs and humans, however, the A_2b_ receptor has also been reported to dilate human coronary arteries [8], and possibly rat coronary arteries [6]. In the guinea pig, A_2b_ appears to predominate in the thoracic aorta to induce relaxation [9] and both A_2a_ and A_2b_ in the rat [10–12]. In addition, there is ongoing debate on the relative importance of an intact endothelium to adenosine relaxation in these vessels, and the role of nitric oxide (NO) and interplay between voltage-dependent transmembrane Na^+^, K^+^ and Ca^2+^ fluxes and signalling pathways. In the thoracic aorta, adenosine relaxation has been reported to be fully dependent [10, 13], partially dependent [14–17] or not dependent on the presence of an intact endothelium [10, 18–20]. Adenosine vasodilation has also been linked to A_1_ and A_2a_ receptor activation of endothelial production of NO and prostanoids [21], hyperpolarising factors [4], and a complex interplay between endothelial and smooth muscle mitochondrial and sarcolemmal K_ATP_ channels [16, 22, 23], and Na^+^/K^+^ ATPase activation [4, 24].

The aim of the present study was to investigate adenosine relaxation in intact versus denuded rat thoracic aortic rings, and examine the effect of inhibitors of nitric oxide (NO), prostanoids, K_v_ channels, K_ATP_ channels, and adenosine A_2a_ and A_2b_ receptors. The rat thoracic aorta was chosen because of the ongoing debates about the mechanisms of adenosine relaxation, and its in vivo significance.

## Methods

### Animals

Male Sprague Dawley rats (300–350 g, *n* = 47) were fed *ad libitum* and housed in a 12-h light/dark cycle. On the day of the experiment rats were anaesthetised with Na-thiopentone (100 mg/kg). Animals were treated in accordance with the Guide for the Care and Use of Laboratory Animals published by the US National Institutes of Health (NIH Publication No. 85–23, revised 1996). The James Cook University (JCU) Animal Ethics Committee approval number for the present study was A1535. All other chemicals, drugs and inhibitors including adenosine (A9251 > 99 % purity) were purchased from Sigma Aldrich (Castle Hill, NSW).

### Aortic ring preparation and organ bath tension measurements

The thoracic cavity of anesthetized rats was opened and the thoracic aorta was harvested and placed in a modified ice-cold solution of Krebs-Henseleit (118 mM NaCl, 4.7 mM KCl, 1.2 mM Na_2_PO_4_, 0.5 mM MgCl_2_, 1.12 mM CaCl_2_, 25 mM NaHCO_3_, 0.03 mM EDTA) pH 7.4 with 11 mM glucose. The aorta was carefully dissected from surrounding fat and connective tissue and cut into short transverse segments. Intact aortic rings were isolated from each rat and used without further processing. In those studies that required removal of the endothelium, intact rings were denuded by gently rubbing the intimal surface of the vessel segment with a smooth metal probe. Successful removal of the endothelium was assessed by testing the aortic ring for a vasodilatory response to 10 μM acetylcholine (final concentration).

After preparation, intact or denuded aortic rings (3 to 4 mm long) were equilibrated in a standard 10 ml volume organ bath (Radnoti Glass, ADinstruments, NSW, AUS) containing modified Krebs-Henseleit (see above) and continuously bubbled with 95 % O_2_ and 5 % CO_2_ at 37 °C for 15 min (zero tension). The rings were vertically mounted on small stainless steel triangles, stirrups and connected to an isometric force transducer (PANLAB, distributed by ADInstruments as MLT 0201/RAD, NSW, AUS) coupled to a computer based data acquisition system (PowerLab, ADInstruments) and data recording software LabChart 7 (ADInstruments Pty Ltd., Castle Hill, Australia).

The ring tension was manually adjusted to 1.5 g and equilibrated for 60 min. A tension of 1.5 g was chosen from the literature for thoracic aortic rings [25, 26] and preliminary studies verified this tension. During equilibration, the solution was changed in 15 min intervals. The aortic rings were then washed with freshly prepared Krebs Henseleit buffer pH 7.4 and the tension was readjusted to 1.5 g tension. Each preparation was sub-maximally contracted using 3 μl of 0.1 mM NE (0.3 μM final concentration) [27, 28]. Those aortic rings that failed to contract were discarded. Ten microliters of 10 mM acetylcholine (10 μM final concentration) was applied to confirm the presence or absence of an intact endothelium in all preparations. Acetylcholine will induce rapid relaxation of precontracted rings if the endothelium is intact and if the endothelium is removed (or denuded) the rings will remain in contracted state [19]. Aortic rings were considered intact if the relaxation induced by 10 μM ACh was greater than 80 %, and the aortic ring was assumed denuded if relaxation was less than 10 %.

Rings were contracted at least two more times before each experiment until a reproducible contractile response was obtained. Ten to 15 min after this state was achieved the experiment was commenced because preliminary studies showed that the increase in tension and plateau from 0.3 μM of NE was reached at 10 min and remained at this plateau level for over 60 min, the time course of each experiment.

### Adenosine relaxation in intact and denuded rat aortic rings

Adenosine was added into the oxygenated organ bath containing Krebs-Henseleit solution to obtain 1, 5, 10, 50, 100, 500 and 1000 μM adenosine concentrations. The change in tension of pre-contracted intact or denuded rings was measured. The inhibitors used in this study were incubated in organ bath 20–30 min before NE was administered followed by adenosine incremental administration. These included 1) 100 μM N^G^-nitro-L-arginine Methyl Ester (L-NAME) (nitric oxide synthetase inhibitor) and 10 μM indomethacin (cyclooxygenase or prostaglandin inhibitor e.g. prostacyclin). NO and prostacyclin are two major endothelial derived relaxation factors (EDRF), and the inhibitors were only applied in endothelium intact aortic rings, and 2) 1 mM 4-aminopyridine (4-AP) (Non-selective voltage-dependent *K*^+^-channel blocker of the Kv1 to Kv4 families rather than Kv7 channels) [29–31], 10 μM glibenclamide (Non-selective SarcK_ATP_ channel blocker) [32, 33] and 1 mM 5-hydroxydecanoate (5-HD) (MitoK_ATP_ channel blocker) [34], and Na^+^/K^+^-ATPase inhibitor (100 μM ouabain) [24]. These inhibitors were applied to intact endothelium rings in the presence of L-NAME and indomethacin, and without the presence of L-NAME and indomethacin in denuded aortic rings. The adenosine A_2a_ receptor inhibitor was 100 μM 8-(3-chlorostyryl) caffeine (CSC) [35, 36], and the A_2b_ receptor inhibitor was 10 μM 8-(4-(4-(4-chlorobenzyl)piperazine-1-sulfonyl)phenyl)-1-propylxanthine (PSB-0788) [37]. In rat striatal membranes, these antagonists have reported K_i_ values of 24 nM for CSC [38] and 0.393 nM for PSB-0788 [37], and the micromolar concentrations used in the present study were based on previous published studies [39–41]. The inhibitors were applied in endothelium intact and denuded aortic rings in an oxygenated medium. At the end of each experiment, the rings were tested for viability (or patency) by being maximally dilated with 100 μM papaverine, and relaxation was expressed as percentage of maximal relaxation to papaverine [24, 42].

### Statistics

Values are expressed as mean ± SEM. Eight animals (*n* = 8) were used for each group for seven measurement points using ANOVA analysis, and the number of rats was selected from a priori G-power analysis to achieve a level of 1.0. All data was tested for normality using *Kolmogorov-Smirnov* test. Relaxation responses to adenosine were analysed for homogeneity of variances followed by two-way ANOVA coupled with the *Bonferroni* post-hoc test for individual data point comparisons. The alpha level of significance for all experiments was set at *p* < 0.05.

## Results

### Intact versus denuded aortic rings

**Fig. 1:**
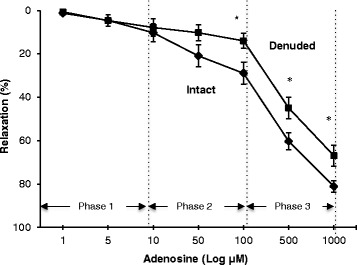
Concentration response curves to adenosine in intact and denuded isolated rat aortic rings. Relaxation is expressed as percent of maximal relaxation to 100 μM papaverine. Points represent mean ± S.E.M of aortic rings from a total of seven animals. *P < 0.05 statistical difference in responses between the intact and denuded rings. Symbols (♦) Intact rings (■) Denuded rings

In endothelium-intact rat aortic rings, adenosine led to 10, 21, 29, 60 and 81 % relaxation at 10, 50, 100, 500, and 1000 μM adenosine concentrations respectively (Fig. 1). Adenosine relaxation in intact rings occurred in three linear phases (log scale); 0.96 % per μM from 1 to 10 μM adenosine (Phase 1), 0.2 % per μM from 10 to 100 μM adenosine (Phase 2), and 0.06 % per μM from 100 to 1000 μM (Phase 3). After removing the endothelium, relaxation was reduced to 8, 10, 14, 45 and 67 % respectively, and was significant from 100 to 1000 μM. In denuded rings, adenosine relaxation was 0.72 % per μM from 1 to 10 μM adenosine, 0.07 % per μM from 10 to 100 μM adenosine (Phase 1), and 0.06 % per μM in Phase 2 from 100 to 1000 μM. Thus endothelial removal of rat aortic rings decreased the responsiveness (defined as % relaxation per μM) to around one-third between 10 and 100 μM, but not in the lower (Phase 1) or higher (Phase 3) ranges (Fig. 1).

### Intact aortic rings

#### Effect of L-NAME and indomethacin

**Fig. 2:**
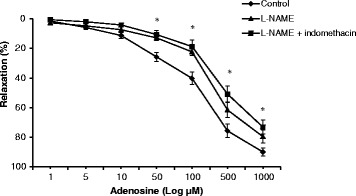
Concentration-response curves to adenosine with and without the presence of L-NAME alone and L-NAME + indomethacin in intact isolated rat aortic rings. Relaxation is expressed as percent of maximal relaxation to 100 μM papaverine. Points represent mean ± S.E.M of aortic rings from a total of eight animals. *P < 0.05 statistically different in the presence of L-NAME alone (▲) and L-NAME + indomethacin (■) compared to control on intact rings (♦)

Figure 2 shows that L-NAME and indomethacin significantly reduced adenosine relaxation at 50 to 1000 μM adenosine. At 50 μm, relaxation decreased from 26 to 11 % or 42 % (11/26 × 100) of the relaxation of intact controls. Thus at 50 μM adenosine 59 % of relaxation was linked to L-NAME and indomethacin inhibition. At 100, 500 and 1000 μM adenosine concentrations, L-NAME and indomethacin contribution to inhibition were 53, 33 and 19 % (Fig. 2). In addition, experiments with L-NAME alone showed a similar inhibition, indicating that indomethacin had little or no significant inhibition (Fig. 2). However, at 500 uM and 1000 uM adenosine there was a small difference of indomethacin from L-NAME but not significant (Fig. 2).

### Effect of K_v_, SarcK_ATP_, MitoK_ATP_ blockers and ouabain on adenosine relaxation

**Fig. 3:**
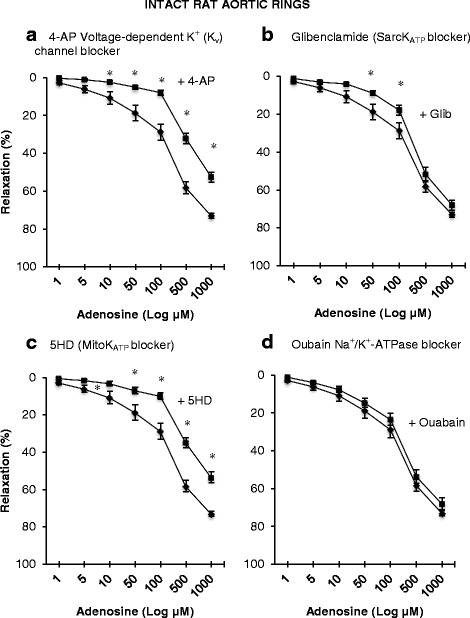
Concentration-response curves to adenosine with and without the presence of some specific ion channel blockers in intact isolated rat aortic rings. **a** In the presence of 1 mM 4-aminopyridine (■). **b** In the presence of 1 mM 5-Hydroxydecanoate (■). **c** In the presence of 10 μM glibenclamide (■). **d** In the presence of 100 μM ouabain (■) compared to controls intact rings (♦). Relaxation is expressed as percent of maximal relaxation to 100 μM papaverine. Points represent mean ± S.E.M of aortic rings from a total of eight animals. *P < 0.05 statistical difference in responses between the presence and the absence of inhibitors on intact rings

The effect of K_v_, sarcK_ATP_, mitoK_ATP_ channels and Na^+^/K^+^-ATPase on adenosine relaxation in intact aortic rings is shown in Fig. 3a–d. In order to eliminate the effect of NO- and prostacyclin-induced relaxation in intact rings, 100 μM L-NAME and 10 μM indomethacin were included in the controls.

#### K_v_ inhibition

Pre-incubating intact rings with 1 mM 4-aminopyridine (4-AP) on adenosine relaxation is shown in Fig. 3a. Percentage relaxation was 2.4, 5.1, 8.0, 32.1 and 52.5 % for 10, 50, 100, 500 and 1000 μM adenosine, respectively. Expressed as a percentage contribution of adenosine relaxation relative to control intact rings, the K_v_ channel was responsible for 78, 73, 72, 58.2 and 28 % for 10, 50, 100, 500 and 1000 μM adenosine respectively, with greater between 10 to 100 μM (Fig. 3a).

#### SarcK_ATP_ and MitoK_ATP_ inhibition

The effect of glibenclamide on adenosine relaxation is shown in Fig. 3b. Glibenclamide was not as striking as 4-AP but significantly decreased adenosine relaxation at 50 and 100 μM adenosine. The contribution of sarcK_ATP_ channel to adenosine relaxation was 63, 53 and 38 % at 10, 50 and 100 μM adenosine (Fig. 3b). MitoK_ATP_ inhibitor, 5-hydroxydecanoate (5-HD), significantly led to a wider range of inhibition of adenosine relaxation compared to glibenclamide from 10 to 1000 μM, but the differences between the two blockers were not significant (Fig. 3c). The contribution of mitoK_ATP_ channel to adenosine relaxation was 70, 63, 65, 40 and 27 % at 10, 50, 100, 500 and 1000 μM adenosine level (Fig. 3c).

#### Na^+^/K^+^-ATPase inhibition

Figure 3d showed that ouabain did not significantly change the inhibition produced by L-NAME and indomethacin in adenosine-induced relaxation at any given concentration, indicating that Na^+^/K^+^-ATPase contributed little extra to adenosine relaxation in endothelium intact aortic rings.

### Effect of A_2a_ and A_2b_ blockers in intact and denuded aortic rings

#### Intact rings

**Fig. 4:**
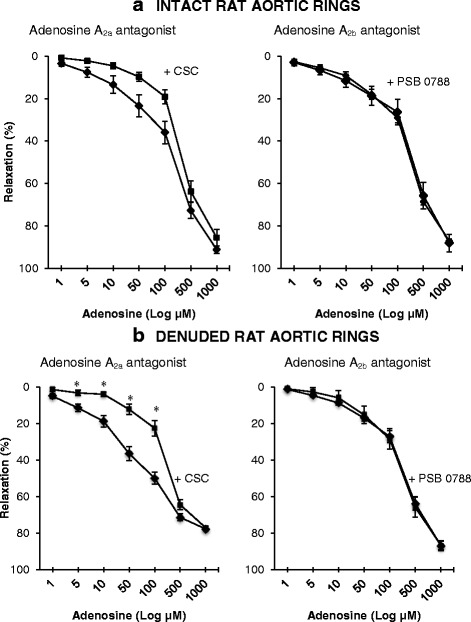
Concentration-response curves to adenosine with and without the presence of adenosine A_2ab_ receptor blockers in intact (**a**) and denuded (**b**) isolated rat aortic rings. In the presence of 100 μM 8-(3-Chlorostyryl) caffeine (■) or 10 μM PSB 0788 (■). Controls (intact and denuded rings) (♦). Relaxation is expressed as percent of maximal relaxation to 100 μM papaverine. Points represent mean ± S.E.M of aortic rings from a total of eight animals. *P < 0.05 statistical difference in responses between the presence and the absence of inhibitors on intact rings

L-NAME and indomethacin were not included in this experiment because it has been reported that NO or prostacyclin release are linked to adenosine A_2a,b_ receptor activation [43]. In the absence of any inhibitors, adenosine induced a rate of relaxation of about 10 % for every 50 μM adenosine up to 100 μM, and ~25 % relaxation per 50 μM from 100 to 1000 μM until 90 % full relaxation (Fig. 4a). Pre-incubating intact rings with adenosine A_2a_ receptor inhibitor, CSC, significantly reduced adenosine relaxation between 50 to 100 μM (Fig. 4a). Although greater percentage falls in relaxation occurred at lower adenosine levels (e.g. 5 to 10 μM) these were not significantly different from controls (Fig. 4a). The A_2a_ receptor was responsible for 71, 66, 59 and 47 % adenosine relaxation at 5, 10, 50, and 100 μM adenosine, respectively. In direct contrast, adenosine A_2b_ receptor inhibitor, PSB 0788, did not change relaxation at any adenosine concentration studied (Fig. 4b).

#### Denuded rings

In denuded rat aortic rings, incubation with A_2a_ blocker, CSC, showed a significant reduction of adenosine relaxation from 5 to 100 μM (Fig. 4b). At 5, 10, 50 and 100 μM adenosine, the A_2a_ receptor was responsible for 72, 79, 66 and 55 % reduction in relaxation. Similar to 4-AP and 5-HD, the A_2a_ receptor blocker did not inhibit adenosine relaxation at 500 uM and 1000 uM. In contrast, adenosine A_2b_ blocker, PSB 0788, had no effect to reduce adenosine-induced relaxation (Fig. 4b).

### Effect of K_v_, SarcK_ATP_, MitoK_ATP_ blockers and ouabain on adenosine relaxation in denuded rings

**Fig. 5:**
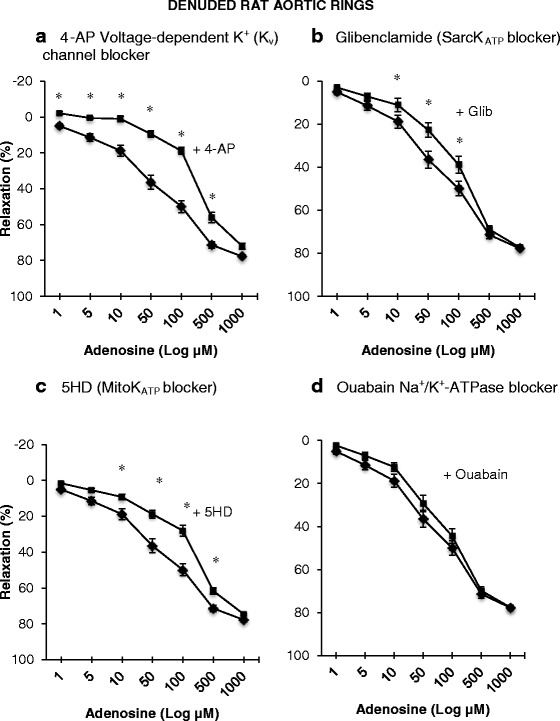
Concentration-response curves to adenosine with and without the presence of some specific ion channel blockers in denuded isolated rat aortic rings. **a** In the presence of 1 mM 4-aminopyridine (■). **b** In the presence of 1 mM 5-Hydroxydecanoate (■). **c** In the presence of 10 μM glibenclamide (■). **d** In the presence of 100 μM ouabain (■). Control denuded rings (♦). Relaxation is expressed as percent of maximal relaxation to 100 μM papaverine. Points represent mean ± S.E.M of aortic rings from a total of eight animals. *P < 0.05 statistical difference in responses between the presence and the absence of inhibitors on denuded rings

In the absence of endothelium and blockers, adenosine relaxed rat aortic rings in a dose-dependent manner and reaching 78 % relaxation at the highest 1000 μM adenosine concentration (Fig. 5). Pre-treatment with 4-AP significantly reduced relaxation from 1 to 500 μM adenosine but not at 1000 μM (Fig. 5a). 4-AP nearly completely abolished adenosine-induced relaxation up to 10 μM adenosine with over 95 % inhibition. At 50, 100 and 500 μM adenosine, the K_v_ channel was responsible for 74 %, 62 %, 21 % of adenosine relaxation (Fig. 5a).

The sarcK_ATP_ channel blocker, glibenclamide, also significantly reduced relaxation at 10, 50 and 100 μM adenosine levels (Fig. 5b) indicating that the SarcK_ATP_ channel was responsible for 41, 38 and 22 % of adenosine relaxation, respectively. Mitochondrial K_ATP_ blocker, 5-HD, significantly reduced relaxation over a wider range than glibenclamide similar to intact rings (Figs. 3b, c and 5b, c). The greatest effect of 5-HD was found at 10 to 100 μM. The contributions of the mitoK_ATP_ channel to adenosine relaxation were 51, 48, 44 and 14 % at 10, 50, 100 and 500 uM adenosine levels, respectively. The Na^+^/K^+^-ATPase channel blocker ouabain, as in intact aortic rings, showed no significant effects to reduce adenosine-induced vasodilation at any given adenosine level (Fig. 5d).

## Discussion

Despite decades of investigation, the mechanisms of adenosine relaxation in large elastic arteries such as the rat thoracic aorta, and smaller muscular resistance arterioles are not fully understood [3, 4, 6, 19, 44]. We report in isolated rat thoracic rings that adenosine vasodilation was: 1) triphasic and partially dependent on an intact endothelium, 2) regulated predominately by endothelial NO, not prostanoids, 3) dependent on opening smooth muscle K_v_, SarcK_ATP_ and MitoK_ATP_ channels, 4) ouabain-insensitive (Na^+^/K^+^ ATPase), and 5) activated by the A_2a_ subtype, not A_2b_. We discuss the possible interplay between these potassium channels and adenosine relaxation in denuded and intact aortic rings, and the in vivo significance.

### Adenosine relaxation involves an NO-dependent pathway

Our study showed that L-NAME significantly reduced relaxation in intact rings and contributed up to 59 % of adenosine relaxation with little or no effect of indomethacin (Fig. 2). In the rat aorta, endothelial NO is believed to induce vasodilation via cGMP- and cAMP-dependent protein kinase mechanisms, and the inhibition of Rho-kinase constrictor activity [45]. The lack of a prostanoid effect in our study was surprising. In 2002, Ray and colleagues showed in an elegant series of studies, using a NO-sensitive electrode, that adenosine relaxation in the rat aorta produced a dose-dependent NO release from the endothelium [46]. They further showed that A_1_-receptor NO release was linked to endothelial prostacyclin release via a common cyclic AMP signalling pathway [21].

In contrast to our study, Ray and colleagues used halothane-O_2_ anesthetized, hypoxic, male 200-250 g Wistar rats, and aortic conduits of 10 mm in length which were longitudinally opened and the NO-sensitive electrode directly in contact with the endothelial surface [21, 46]. Systemic hypoxia in their study was induced using 8 % O_2_ in N_2_ for 5 min prior to aorta harvest, but the group did not specify the pO_2_, pCO_2_ or temperature of their bathing media. This is an interesting contrast, as we harvested the thoracic aorta from normoxic, male 300-350 g Sprague Dawley rats under thiopentone anesthesia, and our isolated intact rings were 3–4 mm in length and fully oxygenated at all times. It is possible that prostanoid production in rat aortic rings is not activated during normoxia but during hypoxia. In 2001, Verma and colleagues also reported in healthy humans that COX-2–selective inhibition did not result in significant changes in endothelial vasodilator responses [47]. Further work is required to examine these differences in different models.

### Role of the endothelium to adenosine relaxation

In the present study, adenosine vasodilation was partially endothelium-dependent (Fig. 1), which is consistent with earlier work of Yen and colleagues [14], Moritoki et al., [15], Headrick and Berne [16] and Rose’Meyer and colleagues [17] in rat and guinea pig thoracic aorta. However, we showed that adenosine relaxation was triphasic (Fig. 1), and that endothelial removal reduced ring relaxation ‘responsiveness’ between 10 to 100 μM adenosine (Phase 2) with little or no change to denuded ring sensitivity from 1 to 10 μM (Phase 1) or from 100 to 1000 μM (Phase 3) compared to intact rings (Fig. 1). To our knowledge, this triphasic nature of adenosine relaxation has not been reported before, and although the underlying mechanisms for the different sensitivities are not known, they appear to involve differential endothelial-smooth muscle sensitivities to endothelial NO production, and smooth muscle A_2a_ receptor and voltage-dependent K_v_ and K_ATP_ channels (see below).

### Role of voltage-dependent K_v_ channels in adenosine relaxation

The 4-AP experiments (~70-95 % inhibition at 5 to 100 μM adenosine) demonstrated that the K_v_ channel has the potential to be a potent activator of adenosine relaxation in rat aortic rings. A similar change in intact and denuded rings (Figs. 3a and 5a) suggests that 4-AP effect was independent of endothelial NO production, and was preferentially activated on vascular smooth muscle (Fig. 3a). Our data support the study of Tammaro and colleagues who reported the presence of smooth muscle K_v_ channels in rat aorta [48], and that of Heapes and Bowles in swine coronary arteries who showed 4-AP-sensitive K^+^ channels in adenosine relaxation [49]. In addition, K_v_ channels have also been widely reported in regulating tone in smaller resistance vessels of cerebral and mesenteric vascular beds [31, 50–52], and in vascular smooth muscle from larger rat pulmonary arteries [53]. In conclusion, our data indicate that adenosine relaxation in isolated NE-precontracted rat aortic rings involved K_v_ channels with higher sensitivities found at lower adenosine levels. Further studies are required using more specific K_v_ channel isoform inhibitors (and agonists), and their membrane voltage dependence on relaxation [54] at low and high adenosine levels.

### Contributions of SarcK_ATP_ and MitoK_ATP_ channels to adenosine relaxation, and A_2a_ receptor activation

We further showed that the SarcK_ATP_ channel contributed to 14 to 63 % of adenosine relaxation up to 100 μM adenosine (Figs. 3b and 5b), and MitoK_ATP_ channels contributed to 22 to 70 % relaxation up to 1000 μM adenosine in intact and denuded aortic rings (Figs. 3c and 5c). The wider range of adenosine inhibition with MitoK_ATP_ channel blocker 5-HD indicates that it shifted the control relaxation curve more to the right than glibenclamide (Figs. 3c and 5c). For example, at 10 and 100 μM adenosine, 5-HD led to 50 % more inhibition than glibenclamide in intact rings (Fig. 5c, b), and 17 and 29 % more inhibition in denuded rings (Fig. 5c, b). This difference may indicate differential contributions of the MitoK_ATP_ and SarcK_ATP_ channel activation to adenosine relaxation, however, 5-HD has been shown to exert effects independent of MitoK_ATP_ channels [55] which may influence that interpretation.

Our glibenclamide data showing significant relaxation reduction (Figs. 3b and 5b), albeit less potent than 5-HD (Figs. 3c and 5c), is in contrast to the study of Husken and colleagues who reported no effect in rat aorta [56]. However, their rings were bathed in a hypoxic, low-glucose medium. Similarly Kemp and Cocks reported lack of a glibenclamide effect in coronary artery rings prepared from cardiac surgery patients [8]. It appears therefore that glibenclamide-sensitive K_ATP_ channel activation and adenosine relaxation is dependent on the state of tissue oxygenation, prior disease states and possibly ischemia.

Furthermore, Kemp and Cocks found that adenosine relaxation in their discarded human coronary artery rings was mediated largely by A_2b_ receptors [8], unlike A_2a_ receptors we found in isolated rat aortic rings (Fig. 4a, b). Adenosine A_2a_ receptor activation and relaxation in rat aortic rings is consistent with the majority of studies in rabbit aorta and mesenteric and coeliac arteries [57], mouse hearts [58], and guinea pig, porcine and bovine coronary arteries [10, 59, 60]. However, Lewis and colleagues reported in Wistar rat isolated aortic preparations that A_2a_ adenosine relaxation was entirely endothelium-dependent [10], not smooth muscle-dependent as we found in the present study (Fig. 4a, b). In rat renal artery, Grbović and colleagues also showed that removal of the endothelium abolished A_2a_ adenosine relaxation, implicating endothelial relaxation factors such as NO for relaxation [42]. These contrasting results may be due to differences in species, age, prior disease state, aortic ring preparation, presence of an endothelium and the bathing media. Another difference may be the type of artery; studying the larger arterial conduits versus smaller arteriolar resistance vessels which have very different functions (see below ‘*Limitations of the Present Study and Future Studies*’). It is noteworthy that Leal and colleagues found that A_2a_ and A_2b_ subtypes were abundant in all three layers of Wistar rat thoracic aorta wall (intima, media, and adventitia) [61], again illustrating the deep complexity of receptor and channel expression in the thoracic aorta.

### Adenosine regulation of relaxation in rat aortic rings: a working hypothesis

Although we did not investigate adenosine relaxation at different oxygenation states and pH, or from hypoxic animals, we propose the following scheme. Adenosine-linked NO production appeared to be a major endothelium-derived relaxing factor in intact rat aortic rings, not prostanoids, which sets the stage for endothelial-smooth muscle coupling. In denuded aortic rings, adenosine appears to activate A_2a_ receptors and trigger downstream opening of K_v_ and K_ATP_ channels located on smooth muscle resulting in membrane hyperpolarization, and relaxation, which may have involved common protein kinase signalling transduction pathways and crosstalk [50, 57, 62–66]. Membrane hyperpolarization of only a few millivolts can lower cytosolic Ca^2+^ via reduced activity of membrane voltage-operated Ca^2+^ channels and reduced myofilament Ca^2+^ sensitivity [67], resulting in smooth muscle relaxation. Partial support for this hypothesis in denuded rings comes from reports showing adenosine activation of K_v_ channels in pig coronary arterioles occurs via cAMP-dependent protein kinase (PKA) activation and vasodilatation [68, 69], and from Kleppisch and Nelson who showed that adenosine A_2a_ (not A_2b_) activation opens K_ATP_ channels via the cAMP/PKA pathway in isolated rabbit mesenteric vascular smooth muscle cells [57]. More recently, Maimon and colleagues showed in skeletal muscle arterioles that PKA signalling varies with pre-exposure to adenosine, and that PKA activation alone was not sufficient to dilate these arterioles, and required other Ca^2+^-dependent mechanisms to facilitate vasodilation to adenosine [66].

Another possible mechanism coupling adenosine A_2a_ receptor to opening K_v_ and K_ATP_ channels in rat denuded aorta rings may be via mitochondrial production of H_2_O_2_ [70, 71]. H_2_O_2_ is a highly diffusible and signalling redox intermediate produced during mitochondrial phosphorylation of ADP to ATP, and is believed to trigger Ca^2+^ sparks that activate protein kinase pathways and adenosine relaxation [72, 73]. Dick and colleagues reported that H_2_O_2_ activated K_v_ channels and led to coronary vasodilation along with increases in myocardial metabolism [72], and Sharifi-Saniani and colleagues showed that adenosine A_2a_ receptor activation in mouse aorta during reactive hyperemia was coupled to smooth muscle K_ATP_ channels via the production of H_2_O_2_ [73]. It is possible that mitochondrial H_2_O_2_ bursts may also facilitate crosstalk between mito- and sarc-K_ATP_ channels in our model.

Lastly, activation of A_2a_ in rat aortic rings may also have occurred from adenosine’s breakdown metabolite, inosine (via adenosine deaminase), which has recently been shown to be a functional agonist of the A_2a_ receptor [74]. It is possible therefore that adenosine engages A_2a_ receptor to generate initial relaxation followed by a dual agonist-mediated response from inosine to amplify or prolong A_2a_ activation in vivo. While inosine is known to relax aortic rings [75], its dual action with adenosine has only been studied in inflammatory/immune cells [74].

### Limitations of the present study and future studies

While all four major types of K^+^ channels (K_v_, K_ATP_, K_IR_ and K_Ca_) appear to be present in vascular endothelial and smooth muscle cells [50, 52, 64, 71, 76, 77], we limited our study to K_V_ and K_ATP_ channels in intact and denuded aortic rings. We also investigated aortic ring relaxation in a high pO_2_ environment and it would be of interest to investigate the effect of lowering pO_2_ and changing pH. In addition, adenosine receptor characterization may have been more robust with the use of more than one A_2a_ and A_2b_ antagonist at appropriate concentrations. The isolated aortic ring preparation also lacks sympathetic neurohumoral innervations and the vasa vasorum, which makes translation to the intact vessel challenging. The in vivo significance of our results may relate to regulating compliance of the thoracic aorta as part of ventricular-arterial coupling [78–80]. The thoracic aorta and other large arteries are compliance vessels and are continually subjected to different hemodynamic forces such as mechanical stretch due to pulsatile blood flow, and may adjust vascular tone by changing the balance of vasodilating and vasoconstricting factors and neurohumoral mechanisms [78–80]. In contrast, smaller peripheral and coronary arterioles supply vascular beds and regulate flow by changing resistance to maintain adequate tissue oxygenation. Further studies are required to investigate the possible role of adenosine (and possibly inosine) and its various receptor subtypes to regulate compliance versus resistance vessels (including venous capacitance vessels) in different regions and vascular beds in the body.

## Conclusions

It was concluded that adenosine relaxation in NE-precontracted rat thoracic aortic rings was triphasic and partially endothelium-dependent, and involved endothelial NO production with a complex interplay between smooth muscle A_2a_ subtype and voltage-dependent K_v_, SarcK_ATP_ and MitoK_ATP_ channels, but not a prostanoid-dependent pathway.

### Ethics approval and consent to participate

Animals were treated in accordance with the Guide for the Care and Use of Laboratory Animals published by the US National Institutes of Health (NIH Publication No. 85–23, revised 1996). The James Cook University (JCU) Animal Ethics Committee approval number for the present study was A1535.

### Consent for publication

Not Applicable

### Availability of data and materials

The datasets supporting the conclusions of this article can be made available by emailing the authors.

## Abbreviations

NO: nitric oxide
NE: norepinephrine
L-NAME: L-N^G^-Nitroarginine methyl ester
4-AP: 4-aminopyridine
CSC: 8-(3-chlorostyryl) caffeine
PSB-0788: 8-(4-(4-(4-chlorobenzyl)piperazine-1-sulfonyl)phenyl)-1-propylxanthine
SarK_ATP_: sarcolemma K_ATP_
MitoK_ATP_: mitochondrial K_ATP_ channels
5-HD: 5-hydroxydecanoate
ACh: acetylcholine
